# Size-dependent melting point depression of nickel nanoparticles†

**DOI:** 10.1039/d0na00153h

**Published:** 2020-04-21

**Authors:** Alexander van Teijlingen, Sean A. Davis, Simon R. Hall

**Affiliations:** Department of Pure and Applied Chemistry, University of Strathclyde 295 Cathedral Street Glasgow G1 1XL UK; School of Chemistry, University of Bristol Bristol BS8 1TS UK s.a.davis@bristol.ac.uk Simon.Hall@bristol.ac.uk

## Abstract

We investigate the phase-transition behaviour of nickel nanoparticles (3–6 nm) *via* dynamic TEM. The nanoparticles were synthesized within a reverse microemulsion and then monitored *via* dynamic TEM simultaneously while undergoing controlled heating. The size-dependent melting point depression experimentally observed is compared with, and is in good agreement with existing thermodynamic and molecular dynamic predictions.

## Abstract 2

The melting point depression as a function of size has not been determined experimentally (orange line) for nickel before. This figure shows our results compared with molecular dynamic (blue) and thermodynamic (black, red, green) models.

## Graphical abstract

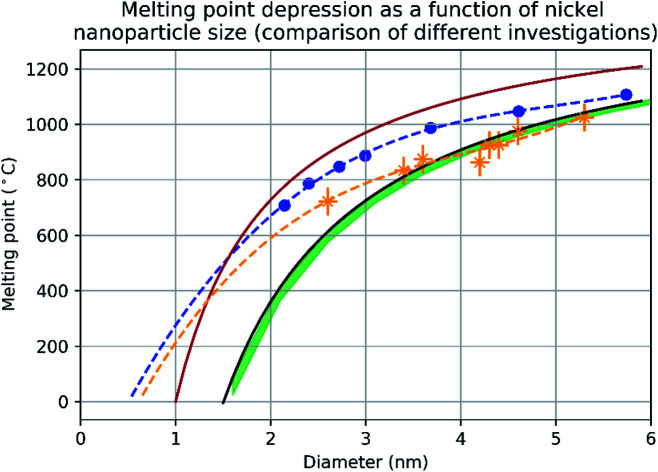

## Introduction

Metal and metal oxide nanoparticles exhibit uniquely size-dependent properties which normally follow an inverse surface area to volume proportionality.^1^ This has a depressive effect on the melting point, and has been shown experimentally for many metals.^2–9^ This effect was first predicted by P. Pawlow in 1909^10^ and demonstrated experimentally in 1954 by M. Takagi.^11^ Buffat *et al.* investigated the melting point depression of spherical gold nanoparticles using the same ED method as Takagi *et al.*, and noted that this method could only determine the range of melting points for an ensemble of particles.^4^ This has long been a standard technology in determining nanoparticle melting points.^12^ It has been purported, however, that measurements of melting points *via* ED becomes increasingly inaccurate as nanoparticle size decreases due to line-broadening.^8^ Determination of melting point depression has also been undertaken through the use of nano-DSC, Lai *et al.* investigated this property of tin nanoparticles, determining the melting point to be when there was an abrupt increase (“jump”) in heat flow required to sustain the increasing temperature.^8^ We apply a similar method as Zhang *et al.* in order to measure individual nanoparticles by direct observation of nanoparticles *via* TEM recognising record melting as an abrupt increase in observed diameter.^13^

## Synthesis

Nickel nitrate hexahydrate (Extra Pure SLR), cyclohexane (≥99.5%) and sodium hydroxide (≥97% NaOH) were purchased from Fisher Scientific. TX-100 (laboratory grade), 1-hexanol (98%) and NaBH_4_ (≥96%) were purchased from Sigma-Aldrich. The reaction procedure was scaled down from the work of Kumar *et al.*^14^ Four syntheses were conducted with different reactant concentrations (ESI Table 1†). The size of the nickel nanoparticles was controlled by varying the molar ratio of water to surfactant (*W*_0_). Each reaction does however produce a range of sizes (ESI Table 1 & ESI Fig. 1–4†), which we find itself useful in this study.

**Fig. 1:**
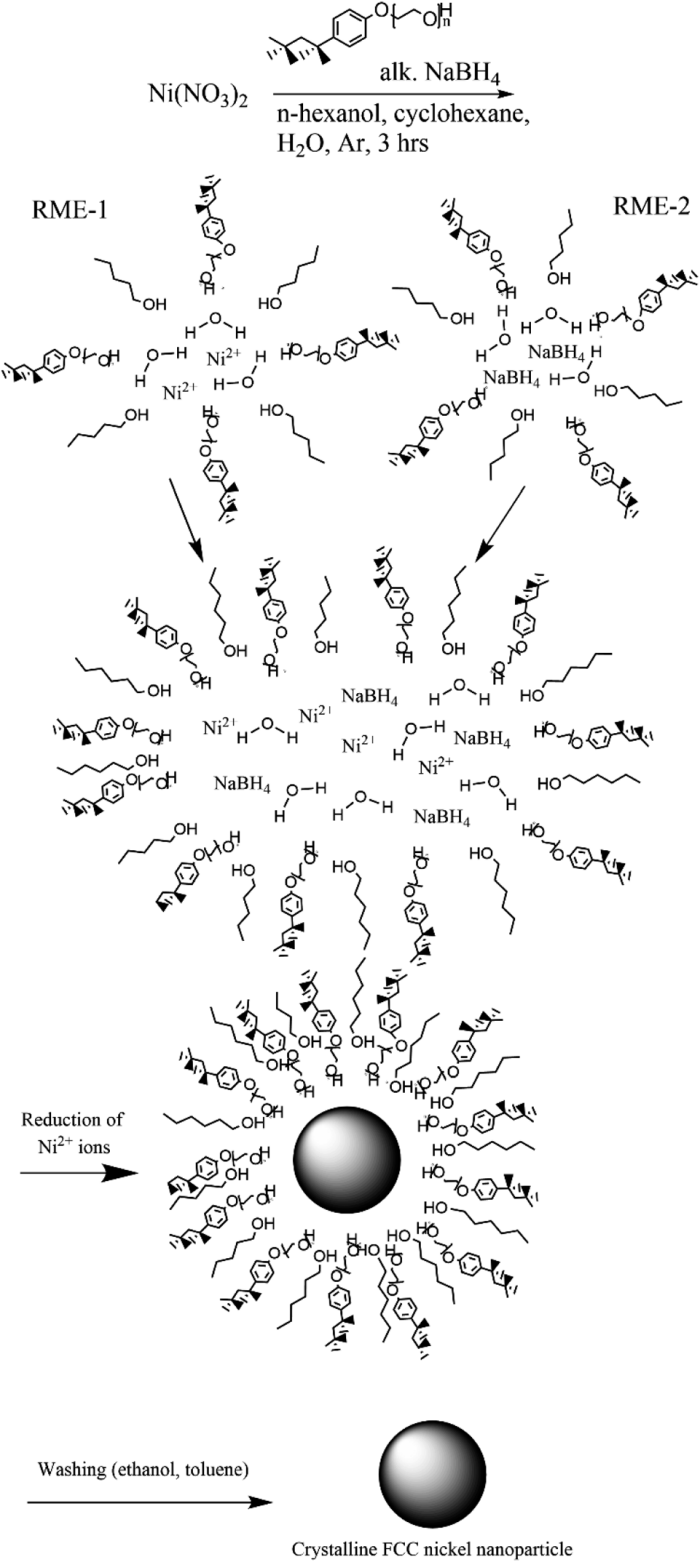
Nickel nanoparticle synthesis procedure consisting of preparation of two reverse microemulsions (TX-100 with cosurfactant 1-hexanol stabilizing the spherical structure), one with nickel(ii) ions within and another with sodium borohydride within.

**Fig. 2:**
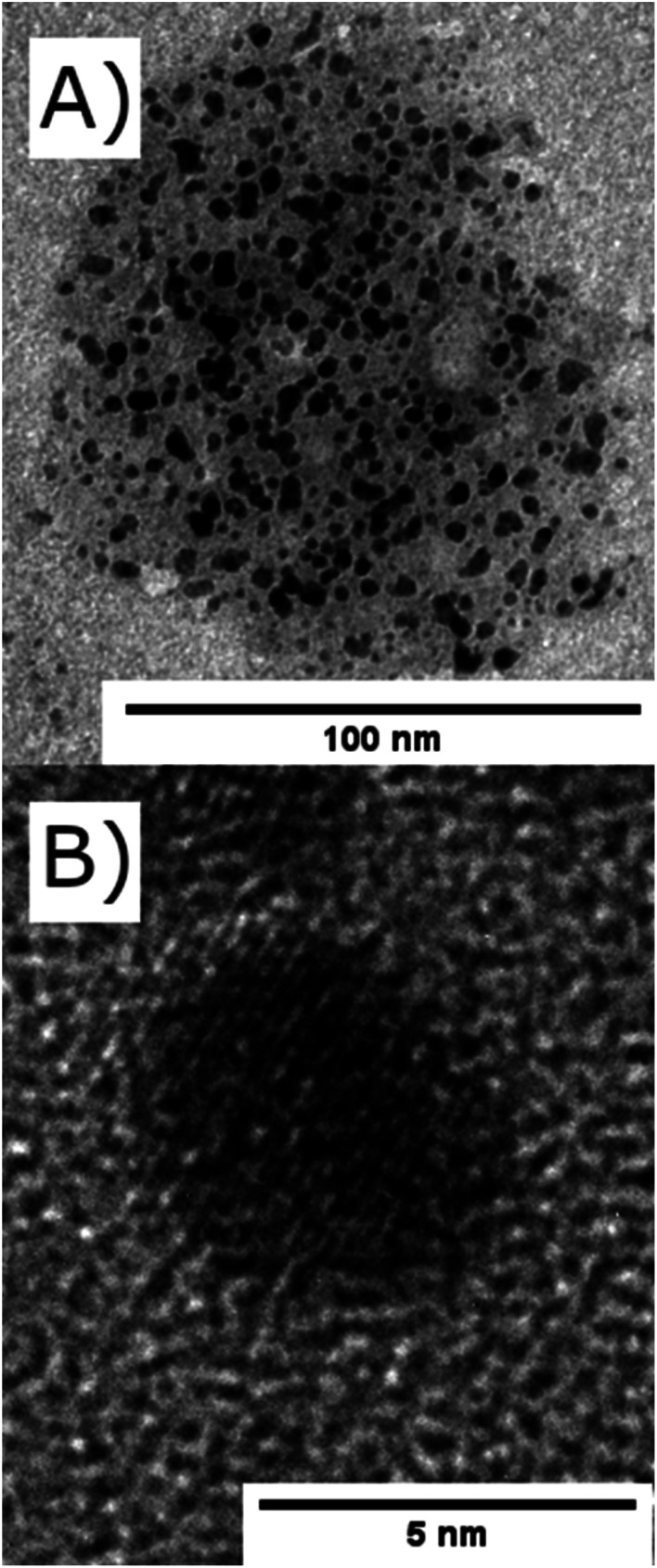
TEM images of (A) nickel nanoparticles encapsulated within a reverse microemulsions of TX-100/1-hexanol (*d* = 100 nm) and (B) a single nickel nanoparticle (*d* = 4.5 nm).

In a typical synthesis (synthesis 1) a 5% (w/v) nickel nitrate solution was prepared by dissolving nickel nitrate hexahydrate (11.25 mg, 38.67 μmol) in water (0.225 mL). A 5% (w/v) alkaline NaBH_4_ solution was prepared in a similar fashion. Two reverse microemulsions (RME-1 and RME-2) were made up of cyclohexane (22.744 mL), 1-hexanol (0.3 mL) and TX-100 (1.731 mL, 2.50 mmol). To RME-1 under stirring 5% (w/v) nickel nitrate solution (0.225 mL) was added so that *W*_0_ was equal to 5 or 1 respectively. To RME-2 under stirring 5% (w/v) alkaline NaBH_4_ (0.225 mL) solution was added. Both reverse microemulsions were left to stir for 30 min to achieve homogeneity after which RME-2 was added dropwise to RME-1 with continuous stirring under an inert (argon) atmosphere. The resulting reverse micro-emulsion was left to stir for 3 hours to allow for nanoparticle growth *via* Ostwald ripening. The nickel nanoparticles were separated *via* centrifugation and washed with ethanol and separately with toluene then dispersed in water (Fig. 2). These reverse microemulsions continually collide and coalesce, mixing their internal constituents – this allows the sodium borohydride to reduce the nickel ions which then grow into a nanoparticle within the microemulsions. The synthesis has been visualized in Fig. 1.

## Melting point characterization

Phase-pure and oxide free nickel nanoparticles on carbon coated copper TEM grids were prepared by drop-casting of a colloidal suspension of nickel nanoparticles in toluene. Using a Gatan heating stage (model number 628) with applied water cooling coupled to a JEOL-2100F TEM, we recorded the melting point of eight different nickel nanoparticles over a temperature range of 700–1100 °C at a rate of 5 °C min^−1^. Nanoparticle diameters were measured only when the video capture showed clearly de-fined edges. The heating stage independently measured the temperature of the sample and therefore heating effects from the electron beam are accounted for in our measurements. Recording the expansion of nanoparticles around the melting point allows the determination of this physical property unambiguously. Nanoparticles were determined to have melted when three requirements had been met:

1. The average diameter of the spherical nanoparticle increased by at least 4.3% (this percentage was chosen as it is the difference in diameter for two equimolar spherical nickel nanoparticles, one a solid and one a liquid with perfect wetting on a substrate, densities 8.908 g cm^−3^ and 7.81 g cm^−3^ respectively).

2. The melting point is clearly identified by a rapid and sustained increase in size as opposed to gradual expansion due to heating of a solid.

3. The diameter of the liquid phase nanoparticle used to deter-mine the melting point does not have overlapping error bars with the diameter measurements of the solid nanoparticle.

**Fig. 3:**
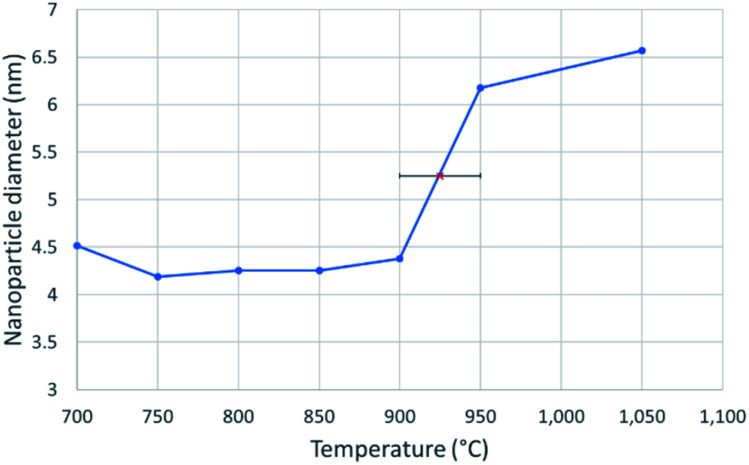
A line graph showing the diameter of a 4.3 nm nickel nanosphere over a temperature range of 700–1050 °C, at intervals of 50 °C. The solid nanoparticle diameter was taken as an average of 5 measurements between 700 °C and 900 °C. The red X in the centre marks the melting point at 925 ± 25 °C, which was determined from the abrupt, sustained and significant increase in nanoparticle diameter.

Images at different temperatures were analysed using the soft-ware package ImageJ2, Fiji distribution^15,16^ by recording the projected TEM image surface area of individual nanoparticles (Fig. 3) and the diameter calculated according to (1).1
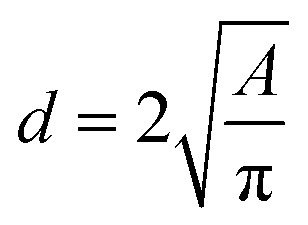


**Fig. 4:**
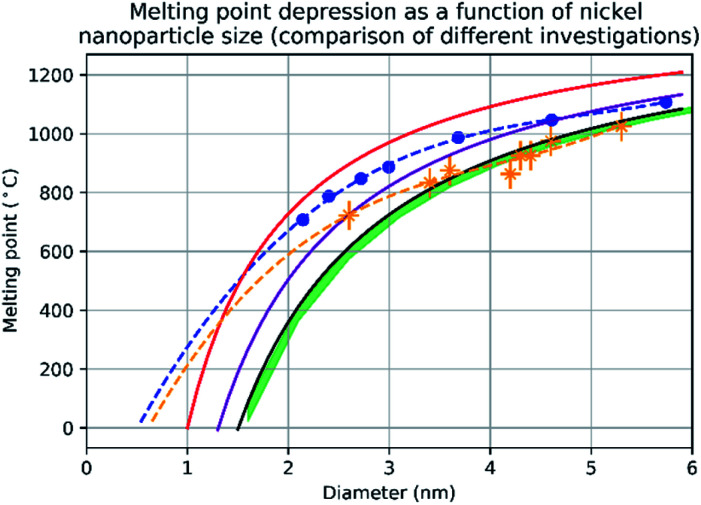
A graph showing the results of evaluating the melting point depression of nickel nanoparticles by four different methods. The red line denotes the melting point of nickel nanoparticles according to the empirical model (eqn (2)) from *d* = 1 nm to *d* = 6 nm with 1 nm increments. The blue trendline and markers plot the melting point results of the molecular dynamics study using the quantum-Sutton-Chen (QSC) force field reported by Qi *et al.*^3^ The purple line denotes values predict by the liquid-drop model^24^ with a *β* value given by Hanszen *et al.*^25^ The black line and markers represent values for the melting point as a function of size predicted by the Gibbs–Thompson (eqn (3)). The green area shows the temperatures between the upper and lower bounds of the liquid nucleation and growth model (eqn (4) and (5)) – this is in very close agreement with the experimental data between 3.5 nm and 5.3 nm, where most of the experimental data points where collected. The orange trendline and markers display the experimental data collected in this study, the vertical error bars show a ±50 °C uncertainty in temperature and the horizontal error bars show a ±0.1 nm uncertainty in diameter.

Fig. 3 shows the change in mean diameter as a function of projected nanoparticle surface area, for a single nickel nanoparticle over increasing temperatures. There is an abrupt increase at the melting point (925 °C ± 25 °C). The melting point was taken as the temperature halfway between the upper and lower points of the increase. This method is similar to the nano-DSC method described by Lai *et al.*^8^ This process was repeated for seven different nickel nanoparticles and the determined melting points plotted in Fig. 4. This experimental data is in good agreement with the MD studies and thermodynamic models also shown in Fig. 4.2
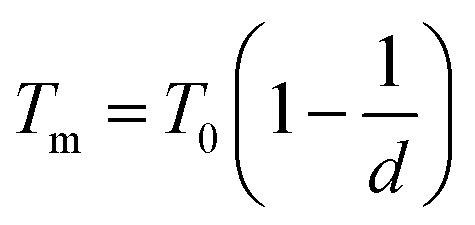


The empirical relationship between diameter and melting point can be described by eqn (2) where *T*_0_ denotes the bulk melting point, *d* the diameter, *T*_m_ the melting point of the nanoparticle and the melting point is inversely related to the diameter.3
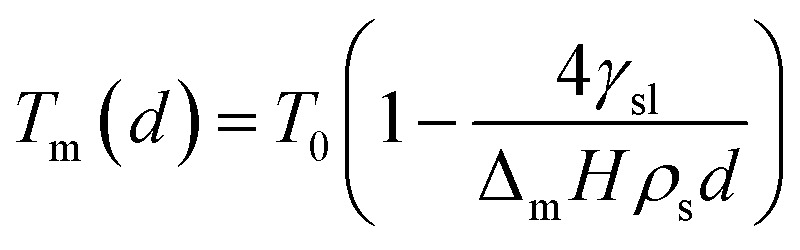


There exist several proposed mechanisms in which melting initialization and propagation in nanomaterials is described as a function of interface energies and the enthalpy of fusion (Δ_m_*H*). One of these models is the Gibbs–Thompson eqn (3), which assumes an isolated spherical nanoparticle situated within it's a liquid of its own type.^7^ While this model has some success in predicting melting point depression in our nanoparticles,^2^ it is generally believed that it is an incomplete model of solid–liquid phase transition in nanoparticles. More sophisticated models, such as the liquid nucleation and growth model (eqn (4) and (5)), which supposes that the liquid phase is initiated at a surface (a discontinuity) and propagates inwards towards the centre. At a critical radius (r*) an unstable equilibrium is formed, where *r* = r*. The temperature at which this is formed marks the upper boundary of the melting range (green area, Fig. 4), while the lower boundary is given by eqn (5).^17^4
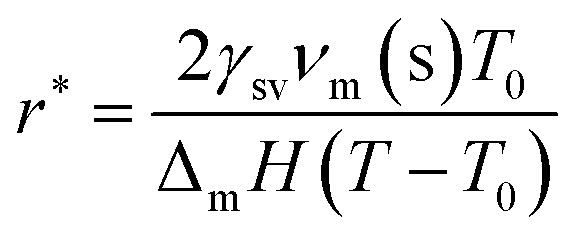
5
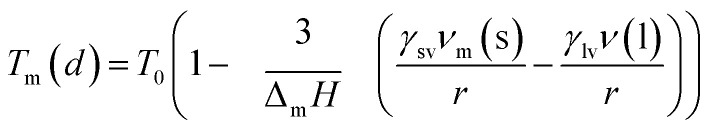


Thermodynamically, for the solid-to-liquid phase transformation to be energetically favourable, the sum of the energies of the newly formed solid–liquid and liquid–vapor interfaces must be less than or equal to the energy of the solid–vapor interface (eqn (6)).

6 *γ*_sv_ ≥ *γ*_sl_ + *γ*_lv_

A key limitation of thermodynamic models is that melting is equivalent to stability such that changes in individual atoms in a system are not considered and therefore cannot reveal the mechanism of the melting process alone. In 2001 Qi *et al.* published a molecular dynamics study calculating the degree of melting point depression in nickel nanoparticles.^3^ By superheating and super-cooling nickel nanoparticles under the QSC potential (eqn (7)), they found the equilibrium melting temperature according to eqn (10). The data presented in Fig. 4 sheds light onto the accuracy of some thermodynamic and molecular dynamic models that have been used to predict the melting point depression as a function of size in nickel, showing that the liquid nucleation and growth model (green area) to be the model that fit best with the experimental data (orange line).7
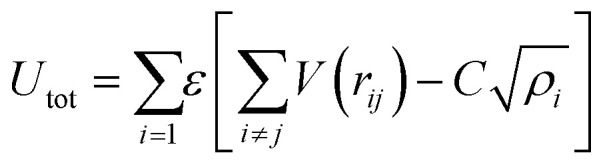
where:8
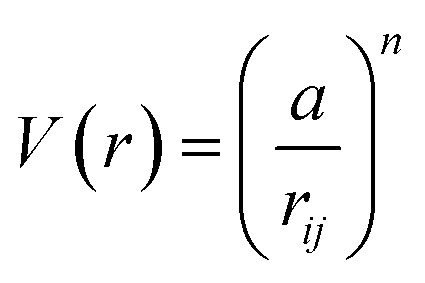
and:9
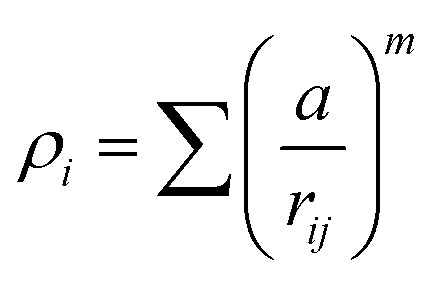
10
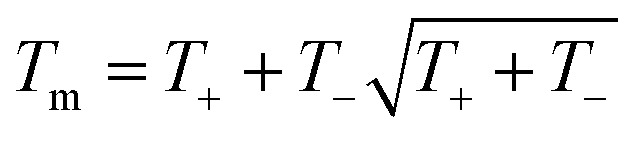


Experimental data presented herein (Fig. 4) have shown that MD studies using the QSC force field, while generally in agreement with the experimental data,^3,18,19^ consistently over-predict the melting points of all sizes of nickel nanoparticles studied – a predictable outcome as their system was shown to determine the melting point of bulk nickel to be 1487 °C (32 °C higher than the real bulk melting point). This QSC force field has previously been used to accurately predict melting point depression in gold,^20^ copper,^6^ platinum^21^ and iron.^22^ It is of note that in each system the bulk melting point of each element was overpredicted. Finally we compared the normalised (Δ*T* = *T*/*T*_0_) and undercooled (Δ*T* = *T*_0_ − *T*) QSC MD study and find that, while closer to the experimental data they over- and under- predict the individual melting points respectively (ESI Fig. 5 and 6†).

## Conclusions

In this study we have synthesized nickel nanoparticles of varying diameter *via* a reverse microemulsion synthesis and for the first time we report experimental studies which complement and validate previous modelling of nickel nanoparticle melting point depression. We have used this method to report a detailed investigation of the melting point depression in nickel nanoparticles. The method could be improved and expanded using alternative TEM grids, such as silicon nitride grids, as they are more stable than copper at elevated temperatures, allowing experiment and modelling to be tested over a larger size range. Future experiments may also include both heating/cooling cycles where the properties of different nanoparticles can be analysed at the 2nd, 3rd, 4th *etc.* cycle of heating as well as their behaviour upon cooling.^23^ With sufficient distribution on the grid, we speculate that this method would be appropriate to measure the properties of completely different nanoparticles simultaneously. Just as we have used it to study different sizes of nickel nanoparticles simultaneously, it could be used to determine melting point depression in magnesium oxide and aluminium oxide simultaneously or the sintering of any two or more different nanoparticles.

## Conflicts of interest

There are no conflicts to declare.

## Supplementary Material

NA-002-D0NA00153H-s001
