# Supplementary material for: Size-dependent melting point depression of nickel nanoparticles
Source: Nanoscale Adv. 2020 Apr 21;2(6):2347–51. doi: 10.1039/d0na00153h (PMC9416969; doi:10.1039/d0na00153h)
Supplement: NA-002-D0NA00153H-s001 [file NA-002-D0NA00153H-s001.pdf]

This journal is © The Royal Society of Chemistry 2020

Table 1. Reaction conditions for synthesis 1 – 4 of different nickel nanoparticles *via* two reverse microemulsions (RME). We find varying nickel ion concentration in the precursor solution did not affect the mode size of nickel nanoparticles predicted but did shift the range of sizes produced.

	RME-1					RME-2							
Synth.	water volume (mL)	Cyhex. volume (ml)	hexanol volume (mL)	TX-100 volume (mL)	Nickel concentration in water (% w/v)	water volume (mL)	Cyhex. volume (ml)	hexanol volume (mL)	TX-100 volume (mL)	NaBH4 concentration in water (% w/v)	Wo	Mode nanoparticle diameter (nm)	Range (nm)
1	0.225	22.7	0.3	1.7	5	0.225	22.7	0.3	1.7	5	5	5	3.6 – 11.6
2	0.225	22.7	0.3	1.7	0.5	0.225	22.7	0.3	1.7	5	5	5	2.8 – 6.7
3	0.225	114.6	1.5	8.7	0.5	0.225	114.6	1.5	8.7	5	1	2	1.9 – 5.5
4	0.45	229.3	3	17.3	0.5	0.45	229.3	3	17.3	5	1	2	1.1 – 3.2

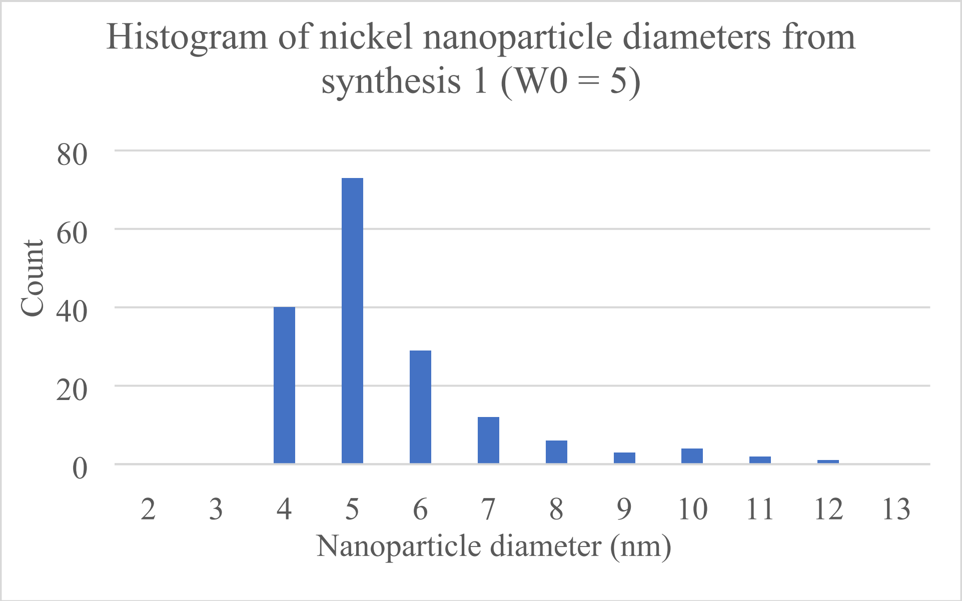

Figure 1. Histogram showing the binned (rounded) values of nickel nanoparticle diameters, determined *via* TEM, for synthesis procedure 1.

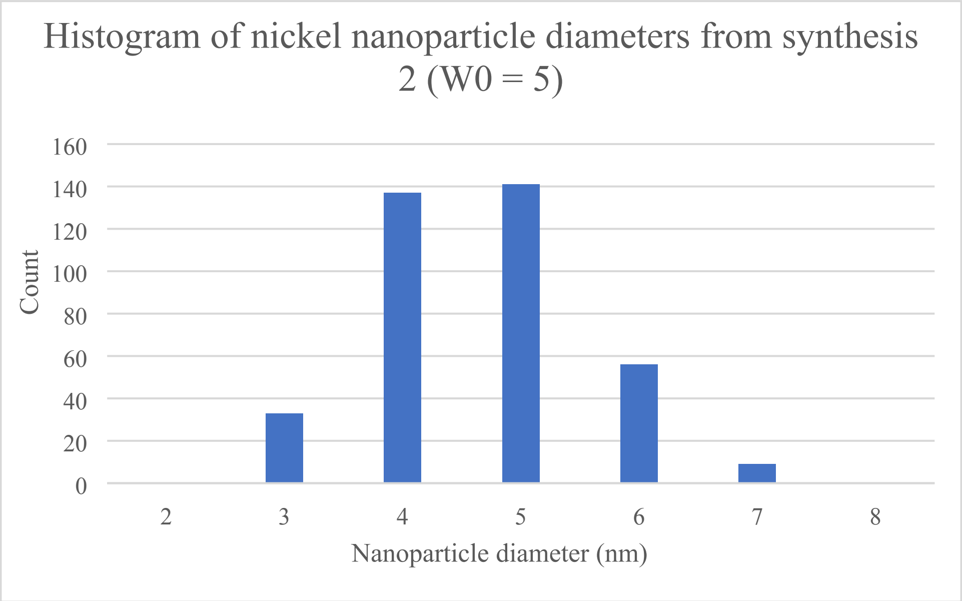

Figure 2. Histogram showing the binned (rounded) values of nickel nanoparticle diameters, determined *via* TEM, for synthesis procedure 2.

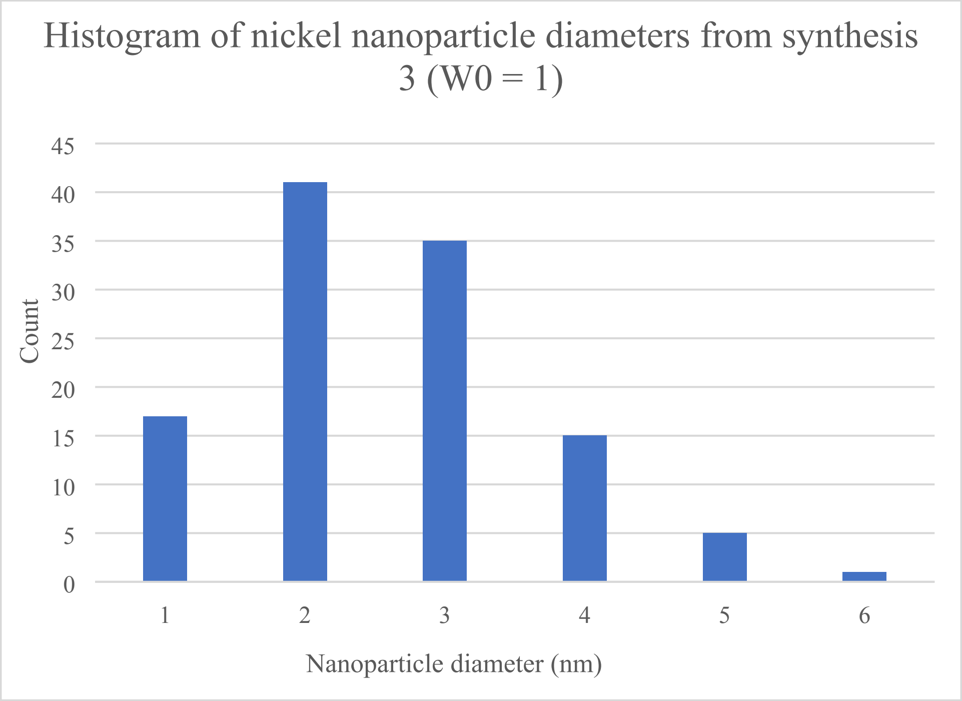

Figure 3. Histogram showing the binned (rounded) values of nickel nanoparticle diameters, determined *via* TEM, for synthesis procedure 3.

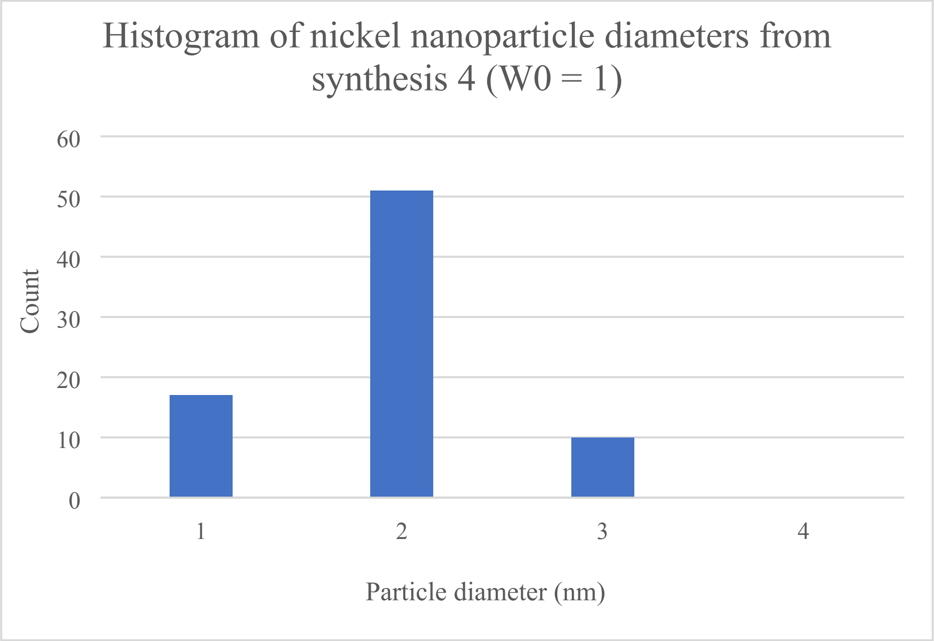

Figure 4. Histogram showing the binned (rounded) values of nickel nanoparticle diameters, determined *via* TEM, for synthesis procedure 4.

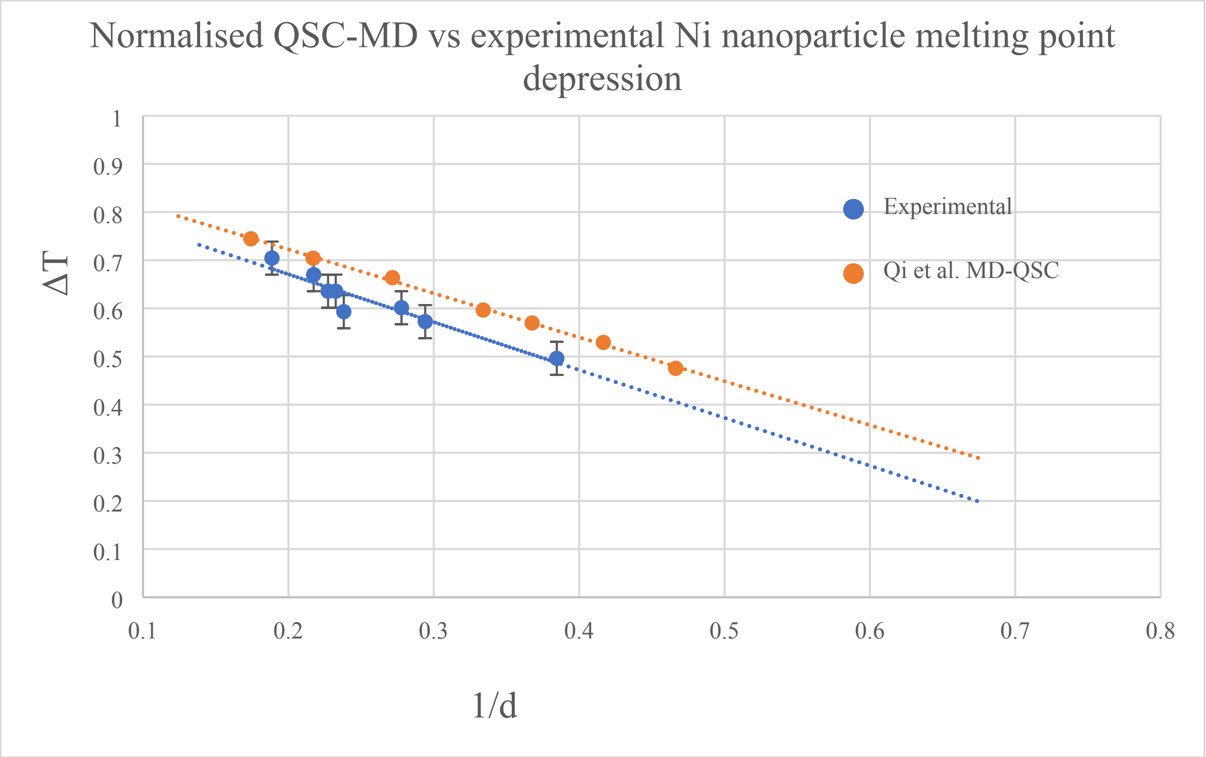

Figure 5. Graph showing a comparison between normalised melting point ($\Delta T = T/T_0$) against the reciprocal of the nanoparticle diameter (d). This graph shows that when we consider the normalised melting points with respect to the experimental melting point and extrapolated melting point from molecular dynamics studies (T_0) that the quantum-Sutton-Chem molecular dynamics model of nickel still overpredicts, outside of the error range, the melting points of most sizes of nickel nanoparticle.

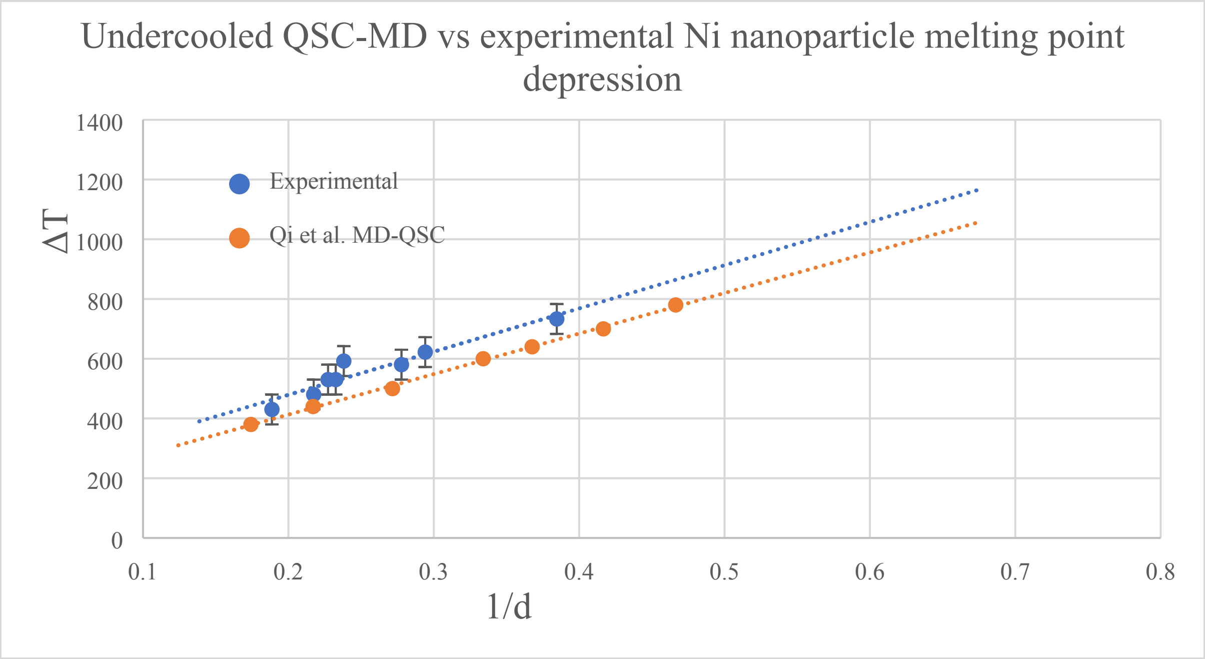

Figure 6. Graph showing a comparison between undercooled melting point ($\Delta T = T_0 - T$) against the reciprocal of the nanoparticle diameter (d). This graph shows that when we consider the undercooled melting points with respect to the experimental melting point and extrapolated melting point from molecular dynamics studies (T_0) that the quantum-Sutton-Chem molecular dynamics model of nickel underpredicts, outside of, but very close to the error range, the melting points of most sizes of nickel nanoparticle.
